# Clinical Use and Molecular Action of Corticosteroids in the Pediatric Age

**DOI:** 10.3390/ijms20020444

**Published:** 2019-01-21

**Authors:** Giovanna Ferrara, Maria Grazia Petrillo, Teresa Giani, Edoardo Marrani, Cesare Filippeschi, Teresa Oranges, Gabriele Simonini, Rolando Cimaz

**Affiliations:** 1 University of Trieste, 34137 Trieste, Italy; 2 Signal Transduction laboratory, NIEHS, NIH, Department of Health and Human Services, Research Triangle Park, Durham, NC 27709, USA; maria.petrillo@nih.gov; 3 Pediatric Rheumatology, Anna Meyer Children University Hospital, 50139 Florence, Italy; teresa.giani@meyer.it (T.G.); gabriele.simonini@unifi.it (G.S.); r.cimaz@meyer.it (R.C.); 4 Department of Medical Biotechnology, University of Siena, 53100 Siena, Italy; 5 University of Florence, 50121 Florence, Italy; edoardo.marrani@unifi.it; 6 Department of Dermatology, Anna Meyer Children’s University Hospital, 50139 Florence, Italy; cesare.filippeschi@meyer.it (C.F.); teresa.oranges@meyer.it (T.O.)

**Keywords:** corticosteroids, systemic corticosteroids, oral steroids, pediatric diseases, side effects, mechanism of actions of glucocorticoids

## Abstract

Corticosteroids are the mainstay of therapy for many pediatric disorders and sometimes are life-saving. Both endogenous and synthetic derivatives diffuse across the cell membrane and, by binding to their cognate glucocorticoid receptor, modulate a variety of physiological functions, such as glucose metabolism, immune homeostasis, organ development, and the endocrine system. However, despite their proved and known efficacy, corticosteroids show a lot of side effects, among which growth retardation is of particular concern and specific for pediatric age. The aim of this review is to discuss the mechanism of action of corticosteroids, and how their genomic effects have both beneficial and adverse consequences. We will focus on the use of corticosteroids in different pediatric subspecialties and most common diseases, analyzing the most recent evidence.

## 1. Introduction

The discovery of corticosteroids more than six decades ago can be considered one of the most important therapeutic revolutions of the last century. Numerous compounds have been synthesized since then, and their use, alone or in combination with other drugs, is crucial for the treatment of many disorders presenting in childhood that sometimes has demonstrated to be life-saving. 

The physiological and pharmacological effects of corticosteroids are mediated through the binding to the glucocorticoid receptor (GR), a protein belonging to the superfamily of nuclear hormone receptors (gene ID: *NR3C1*) [1]. The main characteristic of these nuclear hormone receptors is that they are transcription factors: upon ligand binding, they migrate to the nucleus and interact with specific DNA motifs to modulate transcription of genes. Indeed, the glucocorticoid receptor upon ligand binding translocates to the nucleus of cells and regulates transcription of thousands of genes, thus exerting actions that can be summarized as anti-inflammatory/immunosuppressive, metabolic, and toxic. The anti-inflammatory and immunosuppressive actions of corticosteroids are mainly attributable to the transcription, or repression, of genes expressed in immune cells [2,3]. Corticosteroid exposure, in fact, promotes changes in leukocyte trafficking (into, through and out of the stromal compartments of the bone marrow, thymus, and peripheral tissues), immune cell activation, proliferation and apoptosis, and synthesis/inhibition of mediators of inflammation [4]. Although the anti-inflammatory and immunosuppressive effects are desired, the metabolic and toxic effects are not, because they are responsible for the adverse effects [5]. 

Table 1 summarizes the most frequent diseases for which systemic corticosteroids have been proposed. Despite the widespread clinical use of these agents, several concerns remain about their known side effects. Use of high doses, particularly over a prolonged period of time, is associated with changes in appearance including a “moon-face”, weight gain, centripetal redistribution of fat, muscle wasting, acne, bruising, thinning of the skin, and stretch marks. High doses can also precipitate or exacerbate existing diabetes mellitus and cause hypertension. Prolonged use may impair the physiological process of bone mass accrual and the attainment of peak bone mass leading to an increased risk of osteoporosis and causing the suppression of growth that is crucial for pediatric age. Chronic use of corticosteroids can also lead to cataract formation and glaucoma. Corticosteroids increase the risk of contracting certain infections and reduce the capacity to respond to serious infections. Therefore, clinicians should carefully weigh the potential benefits against the risks before prescribing corticosteroid therapy, especially for long-time treatments [1]. 

The aim of this review is to elucidate the mechanisms through which the corticosteroids work, in order to deal with the main indications of systemic corticosteroid therapies in clinical pediatric practice.

## 2. Corticosteroids: Molecular Basis

Corticosteroids are steroid hormones that are either produced physiologically by vertebrates or are manufactured. Endogenous corticosteroids are synthesized in the adrenal cortex and secreted into the blood to regulate a wide spectrum of physiological systems. All steroid hormones are synthesized from cholesterol and, in humans, the major secretions of the adrenal cortex are cortisol (member of the glucocorticoid family) and aldosterone (a member of the mineralocorticoid family) [6]. Glucocorticoids regulate lipid, glucose and protein metabolism, exert anti-inflammatory/ immunosuppressive actions, and vasoconstrictive effects, whereas mineralocorticoids are the main regulators of electrolyte and water balance [7].

Except for fludrocortisone and desoxycorticosterone acetate, the majority of synthetic corticosteroids mimics the actions of endogenous glucocorticoids. Their clinical potency, indeed, is much higher than cortisol and they do not display mineralocorticoid effects [8]. 

### 2.1. The Glucocorticoid Receptor

The biological actions of endogenous glucocorticoids and its derivatives are mediated via the glucocorticoid receptor (GR). Human GR protein is encoded by the *NR3C1* gene that belongs to the nuclear receptor superfamily of ligand-dependent transcription factors [1]. The human glucocorticoid receptor was cloned in 1985 [9] and its pre-RNA consists of nine exons. The last exon consists of two exons, named 9α and 9β, that give rise to two GR isoforms: the isoform that incorporates exon 9α is called GRα, while the isoform that incorporate exon 9β is called GRβ [10]. GRα and GRβ isoforms show high homology. For the first 727 amino acids they are identical, but then they diverge having GRα 50 more amino acids and GRβ only 15 more amino acids [11]. Despite their 93% sequence similarity, divergent activity has been ascribed to GRα and GRβ. First, the expression of GRα is much greater than the expression of GRβ in most cells and tissues, in both normal and disease states [12]. Moreover, GRα, in the absence of ligand, resides in the cytoplasm of cells and represents the classic receptor that, upon corticosteroid binding, acts as transcription factor. In contrast, the GRβ is mainly localized in the nucleus, does not bind corticosteroids (it only binds the glucocorticoid agonist RU-486), is transcriptionally inactive, and inhibits the transcriptional activity of GRα acting as its dominant negative [10,13,14,15,16]. The ability of the GRβ to inhibit the glucocorticoid actions suggests that changes in the expression levels of this isoform can modulate cellular sensitivity to corticosteroids. It has been shown that TNF-α treatment increases the steady-state levels of the GRβ protein leading to the development of corticosteroid resistance [17,18].

Like other steroid receptors, GR is a modular protein containing an N-terminal regulatory domain (NTD), a central DNA-binding domain (DBD), a hinge region, and a C-terminal ligand-binding domain (LBD) [19]. The NTD domain (the first 421 residues) contains the activation function (AF)-1 domain, a docking site important for the interaction of GR with a variety of gene regulatory proteins such as co-activators, co-repressors, transcription factors, histone modulators, and chromatin remodelers [11,13]. The DBD domain (residues 422–486) is involved in the binding of the receptor to specific DNA sequences termed glucocorticoid-responsive elements (GREs), that are located in the promoter or intronic regions of target genes. The DBD domain contains also sequences that induce receptor dimerization and nuclear translocation [11,13]. The hinge region (residues 487–526), together with the DBD domain, dictates receptor dimerization. Moreover, it confers structural flexibility to the receptor, thus allowing for GR dimers to bind to multiple GREs and regulate gene transcription [11,13]. Finally, the LBD (residues 527–777) at the C-terminus is responsible for hormone-receptor binding that induces activation and nuclear translocation of the GRα [11,13].

During its quiescent state, GRα resides in the cytoplasm in a complex with chaperone heat shock proteins and immunophilins, associated with the cytoplasmic microtubules. The GR-chaperone complexes keep the receptor inactive in a conformation that exposes the LBD and masks the nuclear localization sequence, located adjacent to the DBD and LBD [20]. Upon ligand binding, GR undergoes conformational changes that induce activation of the receptor, dissociation from the chaperone complex and translocation to the nucleus [20]. Once in the nucleus, the receptor binds to DNA regulating the transcription of thousands of genes through a direct or an indirect DNA binding. The direct binding consists of GR homodimers binding to GREs, allowing the receptors to regulate gene transcription in a positive or negative fashion. Moreover, the GR can also directly bind to composite GREs; these regulatory elements contain two adjacent DNA binding sites for two distinct transcription factors. Analogous to the direct binding, composite elements can either promote or repress gene transcription. Alternatively, liganded-GR can regulate, as a monomer, gene transcription in an indirect way, that is via interaction with other transcription factors that bind to their specific responsive elements on DNA [21]. Notably, the indirect DNA binding of the GRα mainly regulates the transrepression of proinflammatory genes whose transcription is induced, for example, by AP-1 (Activating Protein-1), SMAD3, and NF-κB transcription factors. GR associates with and inhibits AP-1-dependent transcription without altering its DNA binding [21,22]. Indeed, DNA-binding inactive mutants of GR are fully capable of inducing AP-1 transrepression [23].

### 2.2. Glucocorticoid-Receptor-Mediated Therapeutic Effects

Corticosteroids are potent immunomodulators. There is no cell of the immune system that is not affected by corticosteroids. Regarding the immune system, corticosteroids attenuate the innate immune response by inhibiting antigen-presenting cell (APC) activation and differentiation, increasing endocytic activity, and reducing secretion of pro-inflammatory cytokines. Moreover, they increase survival and accumulation of neutrophils at inflammatory sites and induce basophil apoptosis [4]. Additionally, the adaptive immunity is also affected by corticosteroid exposure; immature and mature T lymphocytes are susceptible to corticosteroid-induced apoptosis. In addition to inducing apoptosis, corticosteroids also modulate T cell polarization. T cell response is indeed shifted from the Th1 to the Th2 phenotype [4,24] through inhibition of Th1 master regulator factor and an increase in Itk expression, a Tec kinase inducing T helper 2 differentiation via negative regulation of T-bet [25,26]. Also, B lymphocytes are reduced by corticosteroid treatment, although not to the same extent as T cells [27]. Regarding the humoral immune response, corticosteroids increase IgE synthesis, which is driven by the synergistic effects of hormones and IL-4 [28].

The therapeutic effects of corticosteroids on the immune and non-immune cells are mediated by transactivation and transrepression of gene transcription. Corticosteroid target genes that have anti-inflammatory properties are induced by the hormones via GR dimerization and GRE direct binding [29,30]. Many of these genes have well-described anti-inflammatory properties, thereby contributing to the immunomodulatory actions of corticosteroids. Corticosteroids upregulate genes involved in inhibition of chemotaxis, such as adenosine A3 receptor (ADORA), clara cell 10kDa (CC10), thymosin and β4 sulfoxide (CYP1A2) and immunosuppression, such as CD1d, FOXP3, IL-10, decoy receptor for IL-1R, and IL-1 receptor antagonist [31,32,33,34,35,36,37]. Furthermore, corticosteroids induce transcription of inhibitors of pro-inflammatory regulators, such as IκBα, and KLF2 [38,39,40]. 

On the other hand, the anti-inflammatory and immunosuppressive actions of corticosteroids derive from transrepression of inflammatory genes. The mechanism through which the GR inhibits gene transcription is via DNA indirect binding. GR interferes with the activity of “pro-inflammatory” transcription factors, such as AP-1, NF-κB, cAMP response element-binding protein (CREB), interferon regulating factor-3 (IRF-3), nuclear factor of activated T cells (NFAT), Th1-specific T box transcription factor (TBT-1), and GATA3 [41]. Compared to the up-regulation of anti-inflammatory genes, tethering of GR to these transcription factors leads to an amplified phenomenon of immune regulation that results in the repression of chemokines (such as eotaxin, macrophage inflammatory protein, and regulated and normal T cell expressed and secreted (RANTES)), pro-inflammatory cytokines (including TNF, granulocyte macrophage colony stimulating factor, IL-1β, IL-2, IL-3, and IL-6), adhesion molecules, and enzymes (such as nitric oxide and cyclooxygenase 2) [42]. 

The large number of genes that are repressed by corticosteroids via GR tethering made the scientific community believe that this mechanism would be sufficient to exert the corticosteroid-mediated anti-inflammatory effects. In support of this hypothesis many studies have been performed investigating corticosteroid responsiveness using GR^dim/dim^ mice [43]. These mice carry a mutation in the GRα gene that inhibits the dimerization of the receptor along with the ability to bind to GREs, thus allowing us to exclude the GRE-dependent gene transcription. Several reports found that GR^dim^ mutation results in receptors with a reduced or abrogated GRE-mediated gene transactivation [23,44,45]. However, other studies on rat GR^dim/dim^ mutants challenged these findings and demonstrated that GR^dim^ can bind to GREs and other glucocorticoid-binding sequences [46,47]. Indeed, it has been shown that GR^dim^ regulates many genes in common with wild-type GR in addition to genes unique to this receptor mutant [30,47,48]. Moreover, other evidence demonstrates that the GR dimerization and gene transactivation is required to complete and terminate the anti-inflammatory cascade [49,50,51,52].

### 2.3. Glucocorticoid Receptor-Mediated Adverse Effects

The advantages of corticosteroids as immunomodulators are many; however, due to the numerous signaling pathways these drugs regulate, prolonged corticosteroid treatment can also result in multiple adverse effects. 

Relatively little is known about the molecular mechanisms of corticosteroid adverse effects except for the fact that the cushingoid syndrome is related to the endocrine effects of corticosteroids on metabolic tissues. It is known that gene activation, through GR dimerization and direct binding to GREs, up-regulates several enzymes involved in glucose and lipid metabolism (such as phosphoenol pyruvate carboxykinase, tyrosine aminotransferase, and glucose 6-phosphate). Thus, chronic exposure to glucocorticoids results in the manifestation of diabetogenic effects of hypercortisolism [53]. Skeletal muscle, liver, adipose tissue, and pancreas are the main targets affected by cushingoid syndrome. In addition, an increased risk of infection can occur resulting from a prolonged steroid treatment.

High levels of systemic corticosteroids induce not only metabolic dysregulation but also develop glucocorticoid resistance. In a context of glucocorticoid resistance, corticosteroids are unable to dampen and control the immune response. The reduced corticosteroid effect may happen via different ways that include corticosteroid-induced GR downregulation, defective DNA binding or translocation of the receptor, post-translational modifications of the GR [46,54], GR nitrosylation by nitric oxide [55], or increased expression of the GRβ isoform that acts as a dominant negative of GRα [10]. Genetic factors may also contribute to GC resistance [56], such as the occurrence of polymorphisms in GR that may occur within families. For example, there is a marked association of the glucocorticoid receptor 646 C > G polymorphism in resistance to corticosteroids, resulting in severe bronchial asthma [57].

## 3. Rheumatological Disorders 

### 3.1. Juvenile Idiopathic Arthritis

Juvenile idiopathic arthritis (JIA) is the most common rheumatic diseases of infancy, and if untreated, represents an important cause of short and long-term disability.

According to the 2001 classification from the International League Against Arthritis, the definition of JIA is applied to several distinct conditions characterized for a chronic inflammatory process affecting the joints [58]. While most of the patients in Europe and North America present with isolated joint inflammation, a subgroup of patients experiences a more dramatic disease, i.e., the systemic JIA form, in which the systemic features overwhelm joint involvement. 

Due to the heterogeneity of the disease encompassed by the definition of JIA, the use of corticosteroids is different according to the disease subtypes. Patients with systemic JIA have the highest probability of receiving systemic corticosteroids in the first 6 months after diagnosis, when compared to those with other JIA subcategories [59].

As systemic JIA is a potentially life-threatening disease, systemic corticosteroids are widely used to ensure a rapid control of the severe extra-articular features, such as severe anemia, pleural or pericardial involvement, impending macrophage activation syndrome or myocarditis [60]. In these patients, high-dose intravenous corticosteroid therapy represents the standard-of-care treatment and a regimen of 30 mg/kg/day of methylprednisolone (maximum dose 1 g/day) on 1–3 consecutive days is frequently used followed by oral prednisone (1–2 mg/kg/day to a maximum of 60 mg/day) administration.

Despite the role of biologic agents acting against Interleukin 1 (IL-1) and Interleukin 6 (IL-6), many patients with systemic JIA still require corticosteroids to achieve disease remission [61]. In the other subtypes of JIA, the widespread use of nonbiologic and biologic disease modifying anti-rheumatic drugs (DMARDs) have progressively reduced the use of corticosteroids. However, a population-based study by Zamora-Legoff et al. reported the use of systemic corticosteroids in 27% of patients with JIA while intraarticular glucocorticoid injections were used in almost half of patients during the follow-up time [62]. Interestingly, in the studied population systemic JIA patients constituted only 3% of the case of JIA enrolled, suggesting a role of corticosteroids also in the other JIA categories. A course of low-dose oral corticosteroids (i.e., prednisone 5–10 mg/day) is occasionally used as a bridging therapy in patients with severe polyarthritis, shortly after commencement of any DMARDs or biologic agents. More often corticosteroids are administered as intra-articular injections of a long-acting formulation (i.e., Triamcinolone hexacetonide) in the inflamed joints, as this treatment results in a rapid relief of symptoms and in marked functional improvement. The American College of Rheumatology recommends intra-articular corticosteroid injections as first line treatment for juvenile oligoarthritis [63]. In patients with polyarticular JIA multiple intra-articular corticosteroid injections are often associated with DMARDs and/or biologic agents to exert a more rapid anti-inflammatory effect.

While monoarticular JIA is known to respond to intra-articular corticosteroids, in polyarticular disease the addition of methotrexate (MTX) enhances the anti-inflammatory action of corticosteroids and appears to delay the relapse in treated joints as shown by a multicenter study by Ravelli et al. [64].

### 3.2. Pediatric Vasculitis 

Systemic corticosteroids represent the main agents for rapid induction of disease remission in vasculitis. 

The most common systemic vasculitis in childhood is the IgA vasculitis (Henoch-Schönlein purpura), with an estimated incidence of 6–24:100.000 in subjects aged less than 17 years of age. The non-thrombocytopenic palpable purpura with lower extremity predominance is the prominent clinical feature and is variably associated with arthritis, abdominal inflammation, or kidney involvement. Despite a generally favorable prognosis and a spontaneous remission in most of the cases, 11.3% children required hospitalization in a national-based survey in Korea due to a complicated course [65]. In IgA vasculitis, corticosteroids may represent a useful treatment in case of severe gastrointestinal complications, such as massive gastrointestinal bleeding, bowel ischemia or perforation, and intussusception. 

Renal involvement with persistent proteinuria and/or hematuria, is also an indication to corticosteroids treatment, since severity of kidney disease is the main risk factor for an unfavorable long-term prognosis. Indeed, Henoch-Schönlein nephritis represents a relative common cause of end-stage renal failure in developed countries. Oral or intravenous methylprednisolone is commonly used in patients with severe proteinuria and/or impaired glomerular filtration, and with persistent proteinuria, although available studies have an overall low quality of available evidence. 

A Cochrane review, updated in 2015, did not demonstrate the efficacy of corticosteroids in preventing kidney disease in IgA vasculitis [66]. In particular, in children with absent or minor kidney involvement at presentation, treatment with prednisone did not result in any significant reduction of incidence of kidney disease when compared with placebo or supportive treatment. Moreover, two studies assessing the efficacy of prednisone in patients with severe nephropathy (nephrotic syndrome, nephritic syndrome, and reduced kidney function) also failed to show any benefit. The role of corticosteroids is mainly limited to patients with severe kidney disease. Recently a retrospective study by Delbet et al. documented that steroid treatment resulted in a better prognosis in patients with Henoch-Schönlein purpura nephritis (HPN) without crescents, irrespective of baseline proteinuria [67].

Kawasaki Disease (KD) is an acute, self-limited vasculitis syndrome that predominantly affects medium and small sized arteries. Due to coronary arteries involvement and the potential development of coronary artery aneurysms (CAA), this disease is the most common cause of acquired heart disease in children in developed countries.

In KD the first-line treatment, according to the published guidelines, is an association of high dose intravenous immune globulin (IVIG) and aspirin. This treatment has led to a reduced frequency of CAA to 3–6% of all patients. However, a subset of patients with KD (10–20%) is resistant to IVIG infusion and shows a higher incidence of cardiac complications. For years, the use of corticosteroids has been contraindicated in KD since the first retrospective studies suggested a worse outcome in corticosteroid-treated patients [68]. However, a selection bias was likely responsible for such results, as children with more severe disease were more likely to receive corticosteroids.

Recently, the role of corticosteroids in IVIG-resistant KD patients has been reassessed. 

Three recently published meta-analyses independently confirmed the efficacy of corticosteroids in preventing CAA, both as an initial adjunctive treatment in those at high-risk for a poor outcome, and as rescue therapy in IVIG resistant patients [69,70,71]. The safety profile of corticosteroids in these patients was reassuring. Early use of corticosteroids showed an increased efficacy, when compared to a delayed administration. 

Thus, corticosteroids are now considered to be an effective option. However, the optimal administration regimen of corticosteroids remains controversial, as the published studies adopted variable dosing schedule. 

Japanese guidelines for KD suggested the use of prednisolone or intravenous methylprednisolone in patients identified as being at high risk of IVIG resistance [72]. 

While in the Asian population several risk scores have been validated to identify such patients, the absence of such tools in the Caucasian population does not allow for an early identification of children more likely to benefit from corticosteroids [73]. 

In non-Asian populations, Eleftheriou et al. suggested the use of corticosteroids in the following condition: patients resistant to initial IVIg; patients younger than 1 year of age; presenting with marked C-reactive protein elevation, hemophagocytic lymphohistiocytosis, and Kawasaki shock syndrome and patients with coronary and/or peripheral aneurysms with ongoing inflammation [74]. The 2017 guidelines of the American Heart Association suggest the use of methylprednisolone as an additional treatment of non-responders to initial or additional IVIG [75].

### 3.3. Juvenile Dermatomyositis

Juvenile dermatomyositis (JDM) is the most common inflammatory myopathy in the pediatric age. The disease is characterized by muscular involvement, resulting in proximal muscle weakness, elevated muscle enzymes and radiographic or biopsy-proven myositis. Most of the patients also present with the characteristic skin rashes and nail fold capillary changes [76]. Despite its rarity, this disease is a major cause of morbidity and mortality among patients with pediatric rheumatic diseases and corticosteroids still represent the mainstay of therapy in JDM, with almost all patients receiving prednisone at a certain point during the disease course [77]. 

The Single Hub and Access point for paediatric Rheumatology in Europe (SHARE) initiative recommends use of a high dose of corticosteroids (oral or intravenous) as induction regimen in newly diagnosed patients with JDM, in combination with MTX. In the presence of complications, such as severe weakness, involvement of respiratory or swallowing muscles or rapidly progressive lung disease, a high dose intravenous regimen with daily pulse methylprednisolone at a dosage of 20–30 mg/kg/day (maximum daily dose of 1 g) is recommended, followed by either low-dose (prednisone equivalent ≤0.2 mg/kg/day) or moderate-dose (prednisone equivalent > 0.2 to < 1 mg/kg/day) corticosteroids [78]. In a recent survey among German pediatric rheumatologists an intermittent regimen with methylprednisolone (20 mg/kg/day, administered daily for 3 days every 4 weeks for 6 months was also used [79].

The role of corticosteroids in combination with MTX was recently reinforced by a multicenter randomized trial that compared the efficacy and safety of prednisone alone or prednisone plus either methotrexate or cyclosporine in inducing remission in children with new-onset juvenile dermatomyositis. While only half of patients showed an improvement at 6 months if treated with prednisone alone (starting dose 2 mg/kg/day and tapered over 24 months), the percentage increased up to two thirds in the groups treated with the addition of methotrexate or cyclosporine. The safety profile was better in the MTX-treated group [80].

### 3.4. Scleroderma

Scleroderma is a term that encompasses a group of rare inflammatory disorders characterized by fibrosis, in the context of inflammation and vasculopathy. The two main groups are juvenile systemic sclerosis (JSSc) and localized scleroderma (LS), each one with several subtypes. In pediatric age, LS is much common than JSSc. 

JSSc is one of the most severe inflammatory diseases affecting connective tissue due to vasculopathy and fibrosis of internal organs. In a large multicenter cohort of JSSc patients, significant morbidity was reported, with 93% of patients having a multi-systemic disorder and 40% of patients having more than four organs involved. While dermatologic and vascular involvement was almost invariably present, musculoskeletal, gastrointestinal, and pulmonary manifestations were less frequently reported. Renal and cardiac manifestations were rare [81]. Treatment in JSSc is directed to abate the inflammatory process and to reduce the degree of fibrosis and vasculopathy. Currently, no guidelines are available for pediatric JSSc, so treatment is based on a combination of several immunosuppressive agents. 

In contrast to other rheumatic conditions, the use of corticosteroids was contraindicated in these patients following the reported high incidence of renal crisis in adults treated with a high-dose of corticosteroids.

However, the retrospective studies from Stevens et al. reported the use of corticosteroids in almost half of patients, with 15% receiving high dose intravenous pulses [81]. In contrast to data from adult patients, no cases of scleroderma renal crisis were reported in this cohort and in the previous study from Martini et al. [82].

Thus, a judicious use of corticosteroids might be beneficial in selected patients with JSSc, with a tight control of kidney function and blood pressure. 

Localized scleroderma (LS) (also known as morphea) is clearly distinct from systemic sclerosis due to the absence of the Raynaud phenomenon, the lack of specific auto-antibodies patterns, and the absence of involvement of internal organs [83]. However, if untreated, LS might result in permanent morbidity and functional disability. The treatment is directed to halt the inflammatory process before the development of the fibrotic sequelae. Recently, a systematic review and meta-analysis published by our group compared the efficacy of the two main approaches (methotrexate or ultraviolet A (UVA) phototherapy) used to treat these patients. According to our analysis, MTX showed greater efficacy compared with UVA phototherapy, even if MTX-treated patients were more likely to have high-risk subtypes of LS. Interestingly, in the MTX-group almost all patients also received corticosteroids. In a quarter of patients, intravenous route of administration was chosen, with a starting dosage of 30 mg/kg/day of methylprednisolone for at least three days in most of them. In the other patients, oral prednisone at 1 mg/kg/die was the most frequently prescribed dosage [84]. However, corticosteroids were not effective in preventing relapses after discontinuation, as shown by the only randomized study from Zulian et al. Patients were randomized to receive MTX + PDN 1 mg/kg/day or Placebo plus PDN 1 mg/kg/day. While prednisone could induce clinical remission in both groups, two thirds of patients treated with corticosteroids only relapsed after withdrawal; in the MTX-treated group clinical remission was obtained in 67.4% of patients at the 1-year follow-up [85]. The current protocol therefore includes the combination of MTX + prednisone. 

### 3.5. Systemic Lupus Erythematosus 

Systemic lupus erythematosus (SLE) is a chronic autoimmune inflammatory disorder with a very broad spectrum of presenting features, a variety of organ and tissue targets, and unpredictable course and severity within and among individuals. An intriguing interplay between different types of genetic defects and environmental factors seems to contribute to the irreversible loss of immunologic self-tolerance, involving a multitude of cells and cytokines of both the innate and adaptive immune system. The complex, poorly understood, and probably not unique dysregulated physiopathological pathway may explain the failure of so many SLE clinical trials. Corticosteroids still represent the cornerstone of SLE treatment, being one of the few approved medicines. The first documented use of glucocorticoids in SLE dates back to 1950 and, since then, their therapeutic use continues to be fundamental. Indeed, thanks to their complex and wide anti-inflammatory and immunosuppressive action, exerted on both acquired and innate immune compartments, and their rapid onset of action, glucocorticoids are well suited to the treatment of SLE [86,87]. 

The dose and route of administration depend on the type and severity of clinical manifestations. Topical corticosteroids are a mainstay of therapy for the management of cutaneous lupus. Different corticosteroid preparations can be used, from low, to medium, and high potency. The choice should be oriented towards the lowest potency compound able to obtain the best result, used for the shortest amount of time, thus reducing at minimum the possibility of local side effects such as atrophy and telangiectasia. In topical treatments the vehicle is the other important factor to consider: creams and ointments are usually the best choices for the body, while foams, lotions, and solutions are suitable for scalp. Occlusiveness and physician, and patient preference also dictate the type of vehicle chosen [88].

Intralesional steroid injections with 2.5 to 10 mg/mL triamcinolone solution should be limited to refractory cutaneous lesions. The extent and the number of lesions orient towards topical or systemic approach. Low-medium doses of oral corticosteroids (prednisone mean daily dose less than 1 mg/kg/day), alone or, more frequently, in association with others treatments, are usually prescribed to control mild SLE manifestations such as constitutional symptoms, arthralgia, mouth ulcers, or modest changes in blood count, and to maintain a clinical stable disease, helping the prevention of flares in serologically active patients [89].

Higher oral doses (high 2 mg/kg/day) are generally reserved for severe manifestations such as profound cutaneous involvement with bullous or necrotizing lesions, haemolytic anemia, leukopenia, and thrombocytopenia. Intravenous pulse steroids (30 mg/kg, maximum 1000 mg, of methylprednisolone daily for three consecutive days) should be considered in presence of very critical or life-threatening situations e.g., pulmonary haemorrhage, acute lupus central nervous system involvement with psychosis, seizures, movement disorders or myelopathy or significant serositis. High doses of corticosteroids are the base of the induction treatment for clinically significant renal disease. Indeed, lupus nephritis is one of the most serious manifestation of SLE, representing a major risk factor for overall morbidity and mortality and affecting up to 60% of patients. For many years, corticosteroids have dramatically improved patient survival rate, and they still represent the standard-of-care in renal involvement [90]. 

Despite the significant improvement in SLE treatment since glucocorticoids introduction, it appears to be the classic “double-edged sword”. These drugs are responsible for a long list of possible side effects including infections, osteoporosis, metabolic dysfunctions, adverse psychiatric effects, cardiovascular events, cataracts, glaucoma, etc. A judicious use of glucocorticoids, favoring the association with steroid sparing drugs, and focusing on the minimum effective dose, and duration, is an essential part of SLE treatment.

### 3.6. Rheumatic Fever

Acute rheumatic fever is caused by an autoimmune response to throat infection with Streptococcus pyogenes. Cardiac involvement during acute rheumatic fever can result in rheumatic heart disease, which can cause heart failure and premature mortality [91]. 

A recent Cochrane review about the use of anti-inflammatory agents for the treatment of carditis in acute rheumatic fever found no significant differences in the rate of valve disease at one year between corticosteroid-treated and aspirin-treated groups (eight studies, 996 participants). However, these results cannot be considered conclusive because of serious limitations of the data; six of the eight studies evaluated were conducted before 1965s and before the advent of echocardio-graphy [92]. 

One study, involving only 24 patients, showed that patients with acute carditis treated with steroids had a shorter hospital stay than patients treated with salicylates, so the authors recommended the use of steroids in all patients with carditis [93]. 

To date, clinicians conventionally use steroids in the acute phase of carditis for severe cases and for exudative pericarditis. The optimal duration of steroid treatment remains unresolved and should be evaluated according to clinical and echocardiographic findings.

Chorea can affect children with rheumatic fever and is one of the Jones major criteria. Because rheumatic chorea generally subsides spontaneously, pharmacologic therapy is reserved for patients with chorea that prevents independent ambulation, self-feeding, or causes high caloric expenditure and weight loss [94]. In one trial that compared patients treated versus not treated, prednisone-treated patients had a shortened course of chorea (median of 4.0 weeks versus 9.0 weeks) [95]. 

## 4. Uveitis

Uveitis is an inflammatory disorder involving inflammation of the uveal tract. In children, noninfectious, chronic uveitis is a relatively uncommon but serious disease, with the potential for significant long-term complications and possible blindness. The most common causes of uveitis in the pediatric age are idiopathic and related to autoimmune diseases, especially juvenile idiopathic arthritis. Treatment of uveitis in patients with JIA has not been standardized. Topical corticosteroids are very useful and are the first choice in the management of anterior uveitis and scleritis. Systemic corticosteroid therapy is prescribed for severe ocular inflammation, visually significant cystoid macular edema, as well as in sight-threatening complications of posterior uveitis. Prednisone is the most commonly used drug, at the dose of 2 mg/kg/d for remission induction, with a quick taper once inflammation is controlled. Intravenous corticosteroids are sometimes needed in patients who need aggressive management of inflammation, such as those with resistant uveitis, optic nerve involvement, serpiginous choroiditis or panuveitis. The most commonly used drug is methylprednisolone, at 30 mg/kg (maximum dosage 1 g) intravenously over 3 h, for 3 consecutive days or every other day three times in a week [96,97]. 

## 5. Respiratory Disorders 

Systemic corticosteroids are used in various respiratory diseases in children. 

One of the most common uses is in the treatment of acute moderate and severe asthma. In fact, they improve symptoms, oxygenation and pulmonary function and reduce hospital length [98].

Oral prednisolone/prednisone is the most widely used steroid [99]. 

A recent randomized trial including patients aged 1–14 years who presented to the emergency department with acute asthma compared the efficacy of two doses of dexamethasone (0.6 mg/kg/dose) versus a 5-day course of prednisolone/prednisone (1.5 mg/kg/day on day 1, followed by 1 mg/kg/day on days 2–5, conventional treatment) showing the non-inferiority of dexamethasone [100]. 

Corticosteroids are rarely necessary in the long-term treatment of asthma in children and only in severe asthma at the lowest dose necessary to control symptoms. On the contrary oral steroids are widely used to treat preschool children with wheezing for a short course therapy. A recent randomized trial including 624 patients aged 24–72 months showed that oral prednisolone (1 mg/kg/day) had a clear benefit over placebo at reducing the length of hospital stay in children presenting to a pediatric emergency department with virus-associated wheeze and was well tolerated [101]. 

Another common upper airway disease in which corticosteroids, both systemic and nebulized, are widely used and have been demonstrated to be effective is croup. They decrease the need for other drugs, the duration of hospital stays and the need for intubation [102]. 

In cystic fibrosis (CF) airway obstruction and recurrent respiratory infections lead to inflammation, long-term lung damage, respiratory failure and possibly death. Oral corticosteroids at a prednisolone-equivalent dose of 1 to 2 mg/kg/d at alternate days appeared to slow progression of lung disease in CF. However, in the long term, this benefit needs to be weighed against the occurrence of adverse effects [103].

However, with the exception of treatment of allergic bronchopulmonary aspergillosis, in pre-school-aged children with CF systemic corticosteroids are not recommended for routine use, as potential harm outweighs any benefit. Also, inhaled corticosteroids are not recommended for management of CF lung disease, as no clear benefit has been identified [104].

## 6. Gastrointestinal Disorders

Systemic corticosteroids have been used to treat patients with active inflammatory bowel disease (IBD) since the 1950s [105]; however, with the introduction of biologic agents, clinicians had reduced their use to prevent several side effects of corticosteroids. To date, in patients with moderate and severe IBD corticosteroids are still used to induce remission. 

In acute severe ulcerative colitis (ASC) corticosteroids are the gold standard for treatment and are always the first choice [106]. 

A prospective multicenter cohort study in children with acute severe colitis showed that more than 70% of patients responded to a daily oral prednisolone dose of 1–1.5 mg/kg/d (up to 40–60 mg) with no statistical difference in dose between responders and non-responders [107]. 

Of those who were not responding to oral prednisone/prednisolone, approximately two-thirds responded to intravenous corticosteroids (IVCS). IVCS led to clinical improvement in ~70% of pediatric acute severe colitis patients and, in the landmark trial of Truelove and Witts [108], was the most important factor in the reduced mortality rate [109].

Intravenous methylprednisolone 1 mg/kg/day (up to 40 mg/day) once daily in the morning is recommended as the initial treatment at admission; a higher dose of 1.5 mg/kg/day (up to 60 mg/day) in one or two divided daily doses should be reserved for the more severe end of the spectrum and for children who have failed oral steroids prior to admission. A rapid decreased in methylprednisolone to 1 mg/kg/d (40 mg/d) should be employed once response has been observed. Higher doses were not justified according to a recent propensity score analysis in a large pediatric cohort of acute severe colitis [110].

Regarding Crohn’s disease (CD), exclusive enteral nutrition (EEN) is recommended as first-line therapy to induce remission in children with active luminal CD. Oral corticosteroids are recommended for inducing remission in children with moderate to severe active luminal CD only if EEN is not an option. The recommended dose is 1 mg/kg (to a maximum of 40 mg/day) once daily. The rate of response is up to 90%, but after 1 year only 55% are steroid free; around 40% are steroid-dependent and need steroid sparing drugs, while 5–10% are steroid resistant [111]. 

The phenomenon of GC resistance in chronic inflammatory diseases is quite common but still unclear and certainly different from the rare familial condition of primary generalized GC resistance (i.e., Chrousos syndrome). Polymorphisms in genes involved in the nuclear translocation and transcription, transport and/or metabolism of GC have been suggested as possible candidates of observed inter-individual differences in efficacy and toxicity. The best-characterized example is the drug efflux pump P-glycoprotein, a membrane transporter that extrudes GCs from cells, thereby lowering their intracellular concentration [112].

In children with mild to moderate ileo-cecal CD, budesonide may be used as alternative for induction of remission at the dose of 9 mg (up to 12 mg). The drug is taken orally and released in distal small bowel and ascending colon, so that, while acting locally, it has fewer systemic side effects. In distal colonic disease, steroid-based enemas may be used, as in adult patients [113].

Others systemic uses of corticosteroids in gastrointestinal disorders are for rare conditions such as eosinophilic enteritis, celiac crisis and pediatric autoimmune liver diseases. 

Particularly, current therapeutic strategies for autoimmune hepatitis (AIH) consist of an induction with prednisolone (or prednisone) 2 mg/kg/d (maximum 60 mg/day), which is gradually decreased during a period of 4 to 8 weeks, in parallel with the decline of transaminase levels, to a maintenance dose of 2.5 to 5 mg/day. Frequently, azathioprine is included as a steroid-sparing maintenance therapy. In most patients, an 80% decrease of the transaminase levels is achieved in the first 2 months; however complete normalization may take several months [114].

## 7. Haematologic Disorders

The autoimmune cytopenias are a related group of disorders in which differentiated hematopoietic cells are destroyed by the immune system. Single lineage disease is characterized by the production of autoantibodies against red cells (autoimmune hemolytic anemia (AHA)), platelets (autoimmune thrombocytopenia (ITP)), and neutrophils (autoimmune neutropenia (AIN)) whereas multilineage disease may include various combinations of these conditions. For both the primary (idiopathic) and secondary (due to malignancy, systemic autoimmune disease, infectious disease, or a specific drug) forms non-specific immunosuppression with corticosteroids remains the first-line therapy. The only exceptions to steroids are recombinant human granulocyte colony stimulating factor (rhG-CSF) for the immediate treatment of severe infections in AIN, and red cell transfusion, and alkylating agents for cold-type AIHA [115]. 

In fact, corticosteroids are the first-choice treatment in all cases of warm-type AIHA. Initial treatment involves the use of oral prednisone at a dose of 1–2 mg/kg/day; in the case of poor compliance with oral administration, intravenous methylprednisolone can be used (0.8–1.6 mg/kg/day); in severe cases, a higher initial dose may be indicated, i.e., intravenous methylprednisolone 1–2 mg/kg every 6–8 h for 1–3 days [116].

In a recent large national observational French study, first-line steroid therapy was initiated in 92% of patients with AIHA (total duration, 1 to 240 months), and complete remission was obtained at the end of the first month in 58% of cases. Prolonged multimodal therapies (median, 2; range, 1 to 7) were necessary for 45% of patients [117]. 

Steroid therapy is one of the cornerstones in acute ITP. Treatment recommendations take into account the clinical picture and platelet count. It is appropriate to treat the asymptomatic-paucisymptomatic ITP form with platelet count <20 × 10^3^/L because of the high risk of intracranial haemorrhage, and all intermediate form with more petechiae, bruising and mucosal haemorrhages until life-threatening conditions) and severe ITP [118].

## 8. Endocrinological Disorders 

The human adrenal gland cortex produces three classes of steroids: glucocorticoids, mineralocorticoids, and adrenal androgens. Glucocorticoids are essential for the physiological and daily maintenance and regulation of the balance between basal and stress-related body homeostasis. They are also essential for the proper function of almost all organs and tissues. Cortisol is the principal glucocorticoid hormone produced by the adrenal cortex. The daily cortisol production ranges between 5 and 10 mg/m^2^ body surface area. Glucocorticoid replacement remains the cornerstone of treatment for life-threatening endocrinopathies in childhood such as congenital adrenal hyperplasia, Addison disease and secondary hypothalamic-pituitary-adrenal axis deficit. 

Addison disease or adrenal insufficiency is due to underactive adrenal glands associated with a lack of hormones. Adrenal insufficiency may be acute or chronic and may have several causes. Suppression of the hypothalamic-pituitary adrenal axis by exogenous glucocorticoid treatment is the most common cause of an impaired adrenal response in children. 

Acute adrenal insufficiency is a life-threatening disease characterized by hypotension or hypovolemia, low blood sugar, abdominal pain, nausea and vomiting, weakness, lethargy, and confusion. It could be triggered by an intercurrent infection, trauma, surgery, or other stress-causing events. Acute adrenal insufficiency-related shock is a medical emergency often unresponsive to volume replacement and vasoconstrictors and requires prompt recognition and treatment [119].

In the hypotensive patient, rapid restoration of intravascular volume with isotonic sodium chloride containing dextrose is needed. Additional dextrose should be administered as required to treat hypoglycemia. Blood should be drawn to test for cortisol, electrolytes, glucose, and ACTH levels. Simultaneously with the administration of intravenous fluids, stress doses of hydrocortisone should be given. Recommendations for stress doses are empiric and not based on randomized controlled clinical trials. The current recommended stress dose of hydrocortisone is 4 mg/kg or, if weight is not known, 100 mg in children older than 5 years, 50 mg in children aged 2–5 years, and 25 mg in infants (Table 2). The next step is hydrocortisone infusion 2 mg/kg/day until stabilization. Hydrocortisone may be given intramuscularly in case an intravenous access is not available; however intramuscular administration acts more slowly and may be ineffectively absorbed if peripheral perfusion is poor.

Individuals with adrenal insufficiency, irrespective of the etiology (primary, secondary, or tertiary), require long-term glucocorticoid replacement therapy. Oral hydrocortisone is the first line treatment. Nine-12 mg/m^2^/d of oral hydrocortisone is the initial starting dose for individuals with primary adrenal insufficiency (PAI). Patients with central adrenal insufficiency (CAI due to a hypothalamic-pituitary deficit) which is frequently “partial”, usually require a lower dose. Hydrocortisone is administered every 8 h (three times daily), preferably with a larger dose in the morning to mimic physiological cortisol secretion. Careful monitoring of growth and weight gain is mandatory in children on hydrocortisone therapy and doses should be reviewed if linear growth decelerates or weight gain is excessive.

All patients with adrenal insufficiency should be educated about the need to increase their glucocorticoid dose during stress to avoid preventable episodes of adrenal crisis that can be fatal. The dose should be increased between 2 and 10 times the maintenance rate on the basis of stress severity [120,121].

Regarding mineralocorticoids, aldosterone has a key role in water and electrolyte homeostasis. Mineralocorticoid replacement is only necessary in patients with PAI. As the half-life of aldosterone in the circulation is relatively short, the synthetic mineralocorticoid fludrocortisone is used. Fludrocortisone is given as a single morning dose of 0.05–0.2 mg. Treatment surveillance comprises measurement of blood pressure sitting and standing (with a postural drop >20 mmHg indicating under-replacement), and serum sodium, serum potassium and plasma renin concentration.

Dehydroepiandrosterone (DHEA) and DHEA sulphate (DHEAS) are quantitatively the most abundant adrenal steroids. Dehydroepiandrosterone (DHEA) replacement continues to be controversial, with conflicting reports regarding the quality of life. DHEA treatment seems to exert positive effects on mood, sexuality, and subjective health status in adult patients with chronic adrenal insufficiency, especially in women. DHEA treatment may have a role in pubertal females with adrenal insufficiency [122,123]. 

## 9. Neurological Disorders 

Clinical disorders, characterized by episodes of central nervous system demyelination, have been increasingly recognized in children. These episodes consist of acute disseminated encephalomyelitis, optic neuritis, or transverse myelitis and may be either monophasic or relapsing.

Intravenous methylprednisolone was the first-line treatment of choice for these disorders, for both acute attacks and relapses. The decision to treat depends on several factors: clinical features of the attack, with severity of the attack being the most significant factor, the timing of the attack relative to the time of medical evaluation, isolated sensory symptoms and findings on magnetic resonance imaging like gadolinium enhancement, the presence/absence of the T2 lesions at the central nervous system site where the symptoms/signs localize, the T2 lesion number, and T2 lesion volume. The dose is of 1–2 mg/kg/day [124]. 

A recent Cochrane review concluded that, according to moderate quality evidence, corticosteroids given alone do not significantly hasten recovery from Guillain-Barre syndrome nor affect the long-term outcome. On the contrary, according to very low-quality evidence, oral corticosteroids may delay the recovery [125].

The field of neuroimmunology has drastically expanded over the past decade with the recognition and categorization of antineuronal antibodies within the central nervous system. Autoimmune encephalitis is a broad diagnostic category that describes the subacute onset of neuropsychiatric symptoms whose etiology is linked with the clinical sequelae of the inflammatory response to these antibodies. Clinically, a high index of suspicion is required when evaluating children with new onset of neurologic and psychiatric symptoms. The hallmark of autoimmune encephalitis is the progression of symptoms over days to months. Symptoms may include cognitive decline, memory impairment, movement disorders, seizures refractory to conventional therapies, sleep disturbances, language regression, behavior changes, developmental regression, psychosis, anxiety, and obsessive-compulsive disorder symptoms. Early treatment is essential and has been found to be one of the most significant predictors of a favorable outcome. Aimed at reducing inflammation, first-line immunotherapy includes intravenous (IV) steroids, IV immunoglobulin (IVIG), and plasmapheresis. IVCS are typically given as “pulse dosing” at 30 mg/kg up to 1 g to be repeated for 3 to 5 consecutive days. Continued therapy with corticosteroids varies and there are no fixed treatment regimens. Hashimoto encephalitis is classically highly responsive to steroids. IV methylprednisolone is used most often, although oral prednisone is also used. Given the high morbidity associated with high-dose daily oral steroids, IV methylprednisolone used every 2 to 4 weeks may be better tolerated. Clinical improvement usually occurs in 1 to 10 days, although full recovery may take months. The average duration of treatment varies from 6 weeks to several years [126]. 

Historically, corticosteroids have been a treatment option also for idiopathic intracranial hypertension or pseudotumor cerebri syndrome. There are some studies regarding short-term use in the acute setting for patients with severe visual loss, but long-term use should likely be avoided because of their side effects, among which is the rebound intracranial hypertension upon withdrawal [127]. 

Finally, corticosteroids are recommended in idiopathic facial palsy (or Bell’s palsy) in children to reduce inflammation, even though data from the few available trials are conflicting [128]. 

A large multicenter randomized trial is ongoing to allow for the definitive assessment of the efficacy of prednisolone compared with a placebo in the treatment of this condition [129]. 

## 10. Nephrological Disorders

In the field of pediatric nephrology corticosteroids represent cardinal agents in the management of nephrotic syndrome. Nephrotic syndrome (NS) is a clinical diagnosis based on the presence of proteinuria (>40 mg/m^2^/h or >1 g/m^2^/24 h), hypoalbuminemia, edema, and hypercholesterolemia [130].

In the pediatric age despite the fact that many different glomerular disorders can present with a nephrotic syndrome, 90% of patients with idiopathic NS have a histological pattern of minimal change disease, which is known to respond well to corticosteroids. Thus, the response to corticosteroids is adopted in the clinical practice to guide treatment and to limit the necessity of a kidney biopsy. 

Patients are classified as steroid sensitive (SS) if initial treatment with corticosteroids results in complete remission (defined as albumin trace or negative on urine dipstick or proteinuria <4 mg/m^2^ per h or urinary protein: creatinine ratio <200 mg for 3 consecutive days); otherwise they are considered as having steroid-resistant nephrotic syndrome (SRNS) (defined as persistent proteinuria despite 60 mg/m^2^ or 2 mg/kg for 8 weeks) or steroid-dependent (two consecutive relapses occurring while weaning to alternate day steroids or within 2 weeks of steroid discontinuation) disease [131].

In the past a 6-month regimen of high dose of corticosteroids was commonly used, as suggested also by a Cochrane review [132]. In order to reduce the side effects secondary to prolonged high dose of steroid administration, three randomized controlled trials have been recently published to compare the standard regimen to shorter ones [133,134,135].

The three studies did not show any benefits in terms of a risk of relapse or steroid dependence or in the need of additional immunosuppressive agent; thus, a short regimen is nowadays adopted.

The current regimen is based on 4–5 weeks of oral high dose steroids (prednisone 60 mg/m^2^/day or 2 mg/kg/day), followed by a minimum of 6 weeks of alternate day therapy [136].

The response to corticosteroids at the disease onset has also a prognostic value, as children having SR disease show a 50% risk of developing end-stage kidney disease while in children with SS nephrotic syndrome the rate of progression to chronic kidney disease is less than 5%, despite a relative high incidence of relapses [130]. 

The mechanisms underlining steroid resistance in nephrotic syndrome are still under investigation. Extensive application of next-generation sequencing technology has unveiled that genetic mutations in genes responsible for podocyte integrity can be responsible for an intrinsic resistance to corticosteroids and other immunosuppressive agents in a significant proportion of SRNS cases [137]. Furthermore, alterations in genes implicated in the GC mechanism of action have also been shown to modify the individual response to treatment. Polymorphisms in NR3C1, MDR1 and SXR [138,139,140] have been associated with unresponsiveness to GCs in patients with nephrotic syndrome and epigenetic studies have shown that Pad1 and Histone deacetylase-2 expression may predict response to treatment in idiopathic nephrotic syndrome [141,142]. 

Thus, further studies in this field are needed to define a tailored treatment for individual patients, minimizing the risk of un-necessary immunosuppressive treatment and obtaining long-term response.

## 11. Dermatologic Disorders 

Systemic corticosteroids play an important role in pediatric dermatology for their potent immunosuppressive, vasoconstrictive, anti-proliferative, and anti-inflammatory effects. Both short- and long-term corticosteroid treatments may be indicated in cases of inflammatory, autoimmune, and other diseases.

Autoimmune diseases in pediatric dermatology which may be treated with systemic corticosteroids include connective tissue diseases (e.g., lupus erythematosus, dermatomyositis, scleroderma), autoimmune vesciculo-bullous diseases, vitiligo, and alopecia areata.

Also, in cases of vasculitis, panniculitis, auto-inflammatory diseases (e.g., Sweet’s syndrome, pyoderma gangrenosum, SAPHO syndrome), and Stevens-Johnson syndrome (SJS)/toxic epidermal necrolysis (TEN), systemic corticosteroids can be used.

Systemic corticosteroids for acute urticarial and acute exacerbations of chronic spontaneous urticaria may be considered as a short-term treatment (up to 10 days). They are not, however, recommended as a long-term treatment [143].

In the inflammatory skin disease group, atopic dermatitis and psoriasis may represent a therapeutic challenge and it is important for clinicians to know if and when systemic corticosteroids are recommended. Topical corticosteroids are the first-line anti-inflammatory treatment for patients with atopic dermatitis; however systemic corticosteroids (methylprednisolone or equivalent, 0.5 mg/kg/day) may be used for a very short period (up to 1 week) to treat severe acute flares [144,145,146]. Long-term use of oral corticosteroids in patients suffering from atopic dermatitis is not recommended due to the possible severe side effects and the possible rebound phenomenon with the exacerbation of the acute phase of the disease [147]. Due to the potential side effects and the possible rebound phenomenon, oral corticosteroids are not recommended in psoriatic patients and are also not mentioned in the recommendations of an Italian expert group for severe psoriasis in children [148]. Moreover, the use of systemic corticosteroids in patients with plaque psoriasis may induce the development of pustular psoriasis or erythrodermic psoriasis after treatment discontinuation [149].

Alopecia areata (AA) is an autoimmune non-scarring alopecia with a wide spectrum of extension, from small patches of hair loss on the scalp to the loss of all hair on the scalp and body. The natural course of AA is unpredictable, and several treatment options are available for pediatric AA [150]. Corticosteroids play a central role in the management of AA, as topical, intralesional, and systemic treatment [150,151]. Topical corticosteroids (clobetasol propionate 0.05%) are the first-line treatment in cases of acute AA [151]. Intralesional corticosteroid therapy with triamcinolone acetonide is a good option in the case of small patches of hair loss on the scalp or eyebrows but is not commonly used in children <10 years because of the potential pain and the fear of injections [150]. Pulsed oral corticosteroids therapy in children with AA, using oral prednisolone 5 mg/kg/month for 3 months, showed good results without significant side effects. Another available option is the use of oral dexamethasone 5 mg/day twice weekly (2 consecutive days) for children >12 years [151,152]. Some authors have considered the use of methylprednisolone IV 10 mg/kg/day for 3 days a month [151]. In cases of high-dose pulse corticosteroid therapy, the possible systemic side effects require caution, especially in pediatric patients.

In vitiligo, topical corticosteroids are the first-line treatment in the extra-facial areas (barring intertriginous and genital sites) [153]. Pulse therapy (2 consecutive days/week) with betamethasone 0.1 mg/kg for 3 months, followed by tapering of the dose by 1 mg every month over the following 3months, is a possible therapeutic option in cases of progressive generalized disease [154].

The linear IgA bullous dermatosis (LABD) and the dermatitis herpetiformis are the most frequent autoimmune vesciculobullous diseases in childhood, but rarely also other diseases such as bullous pemphigoid and the diseases that belong to the group of pemphigus may be present. In cases of LABD, the first-line treatment is dapsone (0.5–2 mg/kg/day). Short-term prednisone or prednisolone 0.5–1 mg/kg/day may be a good option in the case of incomplete response to dapsone [155,156]. The first-line treatment for dermatitis herpetiformis is a life-long gluten-free diet. However, drugs such as dapsone (0.5–2 mg/kg/day) and systemic corticosteroids (a low level of evidence) can be considered in the inflammatory phase [156,157,158]. In managing bullous pemphigoid and the group of pemphigus, systemic long-term corticosteroids (e.g., prednisolone 1–2 mg/kg/day) are the first-line choice and dapsone, azathioprine, methotrexate, cyclophosphamide, and hydroxychloroquine have been used as steroid sparing agents [156].

In the past, systemic corticosteroids were also considered the main therapy for large, proliferating, obstructive and disfiguring infantile hemangiomas. Systemic propranolol showed more efficacy and less side effects compared with systemic corticosteroids and is now the first-line treatment. Systemic corticosteroids (prednisone or its equivalent, 2–3 mg/kg/day for 1–2 months) can currently be used to treat infantile hemangiomas as a second-line treatment if propranolol is contraindicated [159,160].

In conclusion, despite advances with newer effective immunosuppressive and anti-inflammatory drugs and targeted drugs, such as biologic agents, corticosteroids still remain a cornerstone therapy of several pediatric diseases, especially for acute treatment. They are increasingly being replaced for long time treatments instead, due to the onset of a long list of adverse effects. Clinicians should carefully weigh the potential benefits against the risks before prescribing systemic corticosteroids. 

## Figures and Tables

**Table 1:** The most common indications of systemic corticosteroids in pediatric patients.

Rheumatologic disorders
Juvenile idiopathic arthritis
Pediatric Vasculitis
Dermatomyositis
Scleroderma
Systemic lupus erythematosus
Rheumatic fever
Uveitis
Gastrointestinal disorders
Ulcerative colitis
Crohn disease
Autoimmune hepatitis
Respiratory diseases
Asthma
Viral wheezing
Croup
Cystic fibrosis
Hematological disorders
Autoimmune cytopenia
Endocrinological disorders
Adrenal insufficiency
Neurological disorders
Demyelinating disorders
Autoimmune encephalitis
Idiopathic intracranial hypertension
Idiopathic facial palsy
Nephrological disorders
Nephrotic syndrome
Dermatologic disorders
Chronic urticaria
Atopic dermatitis
Alopecia areata
Vitiligo
IgA linear bullous dermatosis
Herpetiformis dermatitis
Infantile Hemangioma

**Table 2:** Chronic hormone replacement in adrenal insufficiency in children.

Hormone	Dose Range	Daily Doses	Monitoring
Hydrocortisone	15–25 mg/day	Two-three	Clinical assessment
Prednisone	5–7.5 mg/day	Two times	Clinical assessment
Fludrocortisone	0.05–0.2 mg/day	Once	Electrolytes, blood pressure, plasma renin activity
DHEA	25–50 mg/day	Once	Serum DHEAS, androstenedione and free androgen index

DHEA: Dehydroepiandrosterone, DHEAS: DHEA sulphate.
